# hiPSC-derived cortical neurons from ADHD individuals reveal dysregulated glutamatergic development

**DOI:** 10.1038/s41380-025-03213-8

**Published:** 2025-09-19

**Authors:** Rhiannon Victoria McNeill, Zora Schickardt, Franziska Radtke, Robert Blum, Sarah Kittel-Schneider

**Affiliations:** 1 Department of Psychiatry, Psychosomatics and Psychotherapy, University Hospital Würzburg, Würzburg, Germany; 2 Department of Child and Adolescent Psychiatry, Psychotherapy and Psychosomatic Medicine, University Hospital Würzburg, Würzburg, Germany; 3 Department of Neurology, University Hospital Würzburg, Würzburg, Germany; 4 Department of Psychiatry and Neurobehavioural Science; University College Cork, Cork, Ireland; 5 APC Microbiome Ireland, University College Cork, Cork, Ireland

**Keywords:** Neuroscience, Stem cells

## Abstract

Attention-deficit/hyperactivity disorder (ADHD) is a chronic neurodevelopmental disorder characterised by inattention, hyperactivity, and impulsivity, significantly impacting life quality and mortality. It is among the most heritable neuropsychiatric disorders, yet its aetiology remains unclear, hindering the development of novel medications. Previously, research has primarily focused on the dopaminergic and noradrenergic systems using animal models. However, there is growing evidence for a role of the glutamatergic system in ADHD pathomechanisms, and a translational failure between pre-clinical animal models and human clinical trials. We therefore established and characterised a functional cortical neuronal model using human induced pluripotent stem cells (hiPSCs) to investigate glutamatergic development in healthy controls and adult ADHD patients. hiPSCs from healthy controls and ADHD patients showed no difference in their capacity to form cortical neurons (CNs). However, CNs from ADHD patients showed an altered developmental pattern, characterised by changes in extracellular glutamate and decreased transcription of *NEUN*, *PSD95* and *EEAT2*. Moreover, a significant ~50% reduction in *vGLUT2* transcription was observed at multiple time points, suggesting a robust cellular disease endophenotype which might be suitable for future drug screening. Lastly, calcium imaging analysis revealed decreased synaptic signalling strength and frequency, indicating a hypoactive phenotype. In summary, we were able to establish a functional hiPSC-derived cortical neuronal model to investigate ADHD pathomechanisms, which revealed impaired glutamatergic development in ADHD individuals. Our results suggest that the glutamatergic system should also be a target for future drug development.

## Introduction

Attention-deficit/hyperactivity disorder (ADHD) is a relatively common, chronic neurodevelopmental disorder, with symptoms including inattention, hyperactivity and impulsivity [1]. ADHD prevalence in children is estimated at 5.3% [2], and in adults at 2.5% [3]. It is one of the most heritable neurodevelopmental and mental disorders, with a heritability estimate of ~80% [4], of which 16–20% is thought to be due to common genetic variants (e.g. single nucleotide polymorphisms) [5]. ADHD is predominantly a persistent condition which can significantly influence quality of life [6], and is associated with increased mortality rates [7]. Moreover, there are substantial inter-individual differences in patient treatment response [8], and high rates of psychiatric [9] and somatic comorbidity [10].

One of the factors hindering the development of novel ADHD treatments is the heterogeneous and complex aetiology of ADHD [11], which currently remains unclear. Due to the clinical heterogeneity, it is thought that several different pathways may be involved in ADHD pathology [11]. Previous research has demonstrated a role for the dopaminergic and noradrenergic systems, as the dopamine and norepinephrine transporters (DAT and NET) are the main targets of methylphenidate [12], which is currently the first-choice medication for ADHD [13]. However, evidence for an involvement of the prefrontal cortex and glutamatergic neurons has been growing. Meta-analyses have revealed under-activation of the right and bilateral dorsolateral prefrontal cortex (DLPFC) during attention and working memory tasks (respectively) [14, 15]. Recent MRI mega-analyses have additionally reported smaller total cortical surface areas in regions important for executive functions in children with ADHD, along with reduced cortical thickness in areas involved in emotional processing [16]. Longitudinal studies support these findings, providing evidence for delayed development in cortical surface area and thickness (especially in frontal regions) in children with ADHD, suggesting a maturational delay and altered neurodevelopmental trajectory [17–19]. Lastly, the most recent ADHD genome-wide association study (GWAS) found that genes with significantly different predicted gene expression in ADHD were specifically expressed in the frontal cortex, and that there was an enrichment for genes expressed in excitatory neurons [5]. This is supported by a previous study which found that genetic variants in a glutamate gene set was significantly associated with severity of hyperactivity/impulsivity in ADHD patients [20].

A further factor hindering the development of novel therapeutics for ADHD is the lack of translatability between animal models and humans. Currently, approximately ~95% novel candidate psychotropic drugs fail in clinical trials [21]. Although animal models are critical for understanding brain mechanisms and pathology in an in vivo setting, they unfortunately cannot recapitulate the complexity and inherent ‘human-ness’ of mental and neurodevelopmental disorders, underscoring the translational failure between pre-clinical animal testing and clinical trials in humans [22]. Therefore, there is an urgent need for human-based models for drug testing, to help bridge the gap between animals and humans in vivo.

A possible approach for filling this gap, and complementing animal model studies, is the use of human induced pluripotent stem cells (hiPSCs). hiPSCs are generated from somatic cells, and have the potential to differentiate into any cell type of the body [23]. This enables the investigation of typically inaccessible human tissue/cell types (such as viable neurons) which also recapitulate the donor’s unique genotype, an important tenet for highly polygenic and heritable disorders such as ADHD [5]. hiPSC-based modelling is also particularly useful for investigating neurodevelopmental disorders, as the models largely reflect early embryonic development [24]. Consistent with this, in the most recent GWAS genes significantly associated with ADHD were primarily found to be expressed during early embryonic development [5].

hiPSC-based neuropsychiatric disorder modelling has already been successfully used to provide important insights into disorders such as schizophrenia and bipolar disorder; however, there is a distinct lack of studies using hiPSCs for ADHD research [25]. To our knowledge, currently only four ADHD-hiPSC studies have been published. Two of these studies are our own work, using hiPSC-derived dopaminergic neurons to investigate metabolic differences in ADHD patient carriers of parkin (*PARK2*) copy number variables. We found changes in *PARK2* gene and protein expression, ATP production and basal oxygen consumption rate in CNV carriers [26], along with altered transcriptomics [27]. A recent study by Yde Ohki et al. was able to identify proliferation rate alterations in hiPSC-derived neural stem cells derived from male child and adolescent ADHD patients [28]. Lastly, a study using hiPSC-derived telencephalon brain organoids from one healthy control and one ADHD patient reported thinner cortex layers in ADHD organoids [29].

In the current study, we aimed to establish and utilise an hiPSC-derived cortical neuron model to determine whether deficits in glutamatergic function may play a role in ADHD pathogenesis. We generated hiPSC-derived cortical neurons from healthy controls and adult ADHD patients with persistent ADHD. Persistent ADHD is associated with higher severity of ADHD symptoms and poorer clinical outcomes [30], potentially increasing the probability of identifying functional cellular disease endophenotypes, despite patient genetic heterogeneity [31]. Using our model, we were able to identify significant changes to gene expression in glutamatergic neurons and altered neurodevelopmental course, as well as impaired glutamatergic signalling in ADHD patients. Taken together, our results suggest that phenotypic changes in ADHD are conserved in hiPSC-derived cortical neurons, and that the glutamatergic system may play a crucial role in ADHD pathomechanisms.

## Materials and methods

### Participant recruitment

ADHD patients were recruited between 2011–2018 at both the Department of Psychiatry, Psychosomatics and Psychotherapy, University Hospital Würzburg (Germany) and the Department of Psychiatry, Psychosomatic Medicine and Psychotherapy, University Hospital Frankfurt (Germany). Patients were assessed for ADHD by two independent psychiatrists using the DSM-IV or DSM-5 diagnostic criteria and several ADHD-specific self-report questionnaires (WURS-k, ADHS-SB). Healthy controls were recruited primarily from employees or employee relatives, and had no previous or current psychiatric, internal, infectious, or severe neurological disorders. Healthy controls were screened using the Mini-DIPS questionnaire.

### Ethics approval and consent to participate

Informed consent was obtained from all participants. Ethical approval was obtained from the ethics committees at the University of Würzburg (#96/11) and University of Frankfurt (#425/14). All methods were performed in accordance with relevant guidelines and regulations.

### hiPSC generation and maintenance

Fibroblast cells were derived from donor biopsies and hiPSCs generated and quality controlled (QC) as previously described [32, 33]. Briefly, fibroblast cells were reprogrammed using the CytoTune-IPs 2.0 Sendai Reprogramming Kit (Invitrogen). hiPSC clones were checked for the transcript and protein expression of pluripotency-associated markers, absence of the viral genome and mycoplasma, and differentiated into all three germ layers using the Trilineage Differentiation Kit (Miltenyi Biotech). Genetic analyses were also performed to confirm cell line identity and check for reprogramming-induced chromosomal aberrations. hiPSCs were grown on Matrigel™ (Corning)-coated cultureware (diluted according to manufacturer’s instructions) and fed every other day with StemMACS™iPS-BrewXF (Miltenyi Biotech). Cultures were routinely passaged using RELESR™ (Stemcell) when approaching approximately 80% confluency, at a 1:6 ratio. hiPSCs were cultured in an incubator at 37 °C, 5% CO2 and 20% O2. A minimum n = 4 donor hiPSC lines/group (healthy control vs ADHD) was used for experiments, where a biological replicate is defined as the individual donor. The exact sample size for each experiment is given in figure legends, as sometimes cell lines were lost during experiments due to extraneous reasons such as infection or unexpected cell death. Donor demographic data is given in Supplementary Table 1. For previously unpublished hiPSC lines, basic quality control data is provided in Supplementary Fig. 1.

### Neural progenitor cell (NPC) generation and maintenance

Based on QC data, one hiPSC clone per donor was selected for further experiments. hiPSCs were differentiated into NPCs using the Neural Induction kit (Merck), based on modified dual SMAD inhibition (see Supplementary Fig. 1A). Neural rosettes were then selected using the Neural Rosette Selection Reagent (Stemcell) and plated for expansion. NPCs were maintained in Neural Expansion media (NEM; Merck), which consisted of basal media supplemented with 2 mM glutamine and 20 ng/mL FGF-2. NPCs were grown on Matrigel diluted 1:40, and passaged using Accutase (Stemcell) every 3–5 days.

### Cortical neuron differentiation

Cultureware was coated with poly-L-ornithine (10 µg/mL; ThermoFisher) and laminin (10 µg/mL; Merck) prior to seeding. NPCs between passage 3–5 were seeded at a density of 30 000 cells/cm^2^ and allowed to settle for 24 h. NEM was then exchanged for Neuronal Differentiation media (NDM; Merck) consisting of neurobasal media supplemented with 500 µM Dibutyryl cyclic-AMP, 200 µM ascorbic acid and 100 µg/mL Primocin (InvivoGen). For the first two weeks, an 80% media change was performed 3×/week. After this, a 50% media change was performed 2×/week.

### RT-qPCR

Total RNA was extracted using either the RNeasy-Plus Mini or Micro kit (Qiagen) and checked for genomic DNA contamination via *GAPDH* RT-PCR. cDNA synthesis was performed using the iScript cDNA Synthesis Kit (Bio-Rad). Taqman primers (Thermofisher) were used for RT-qPCR (see Supplementary Table 2). RT-qPCR was performed in triplicate for each cDNA sample using a BioRad CFX machine (BioRad). Two reference genes were used, based on stability plots previously generated for different cell types, and relative transcription of target genes was calculated (^ΔΔ^Ct).

### Immunofluorescent (IF) labelling

Cells were cultured in chambered coverslips (Ibidi) and fixed by incubation in 4% paraformaldehyde (Roth) for 15 min. Cells were permeabilised and blocked using PBS + 5% FBS + 1% BSA + 0.2% Triton X-100 for 45 min (Sigma). For receptor proteins, Triton-X was omitted. Cells were then incubated with primary antibody at 4 °C overnight, washed, and incubated with secondary antibody at room temperature for one hour. For details of antibodies see Supplementary Table 3. Liquid mountant containing DAPI (Ibidi) was added to wells and images obtained using the Eclipse Ti2-E epifluorescence microscope (Nikon). Imaging analysis (including cell counting) was performed using the NIS-Elements software (Nikon), and a minimum of 10 images per donor from different XY locations were analysed.

### Calcium imaging

Neurons were incubated in Krebs-Ringer solution (HEPES-buffered) supplemented with 5 μM Oregon Green BAPTA-1 AM (ThermoFisher) for 15 min, washed 3× and allowed to equilibrate in a Stage Top Incubator (Okolab) for 10 min. Time lapse imaging was performed using 3× binning and 50 ms exposure time for a total of 2 min. 3 × 2-min videos were captured per well using different XY locations. For data analysis the open-source software ‘NA3’ was used [34], in order to perform an unbiased spatiotemporal assessment of calcium signals, identifying both local calcium events and signal-close-to-noise activity.

### Western blot

Cells were scrape-harvested into N-PER Extraction Reagent (ThermoFisher) supplemented with Protease Inhibitor Cocktail (Roche) and PhosStop (Roche). Samples were lysed by incubation on ice for 30 min. Samples were then centrifuged at 12 000 rpm for 30 min at 4 °C, and supernatant transferred to fresh tubes. Protein concentration was determined using the ADV02 assay (Cytoskeleton Inc) according to manufacturer’s instructions, and absorbance measured by a Tecan SPARK platereader (Tecan). 20 μg protein was separated on a 4–12% Bolt™ Bis-Tris gel in Bolt™ MOPs buffer (Thermofisher) and electrotransferred onto a Nitrocellulose membrane (pore size 0.45 μM; ThermoFisher). Membranes were blocked for 1 h at room temperature with Intercept® blocking buffer (LI-COR) and incubated with the primary antibody at 4 °C overnight. Membranes were washed 3 × 5 min with PBST and then incubated with the secondary antibody at room temperature for one hour. Membranes were again washed 3 × 5 min with PBST and imaged using a Fusion FX imager (Vilber). Total protein was then stained using Revert™ total protein stain (LI-COR). Protein quantification was performed using Image Studio™ Lite v5.0 software (LI-COR).

### Extracellular Glutamate/Glutamine assay

A total medium change was performed, and 72 h later media was collected. Media was diluted 1:60 with PBS, and the Glutamate/Glutamine-Glo™ assay (Promega) was performed according to manufacturer’s instructions. Luminescence was measured using a Tecan SPARK platereader (Tecan).

### Statistical analyses

For each donor hiPSC line, the mean of ≥2 technical replicates was calculated. These mean values were then used to calculate the means for case-control analyses, to avoid pseudoreplication [35]. Individual donor hiPSC lines are represented as individual dots on all graphs for transparency. Statistical analyses were conducted using Graphpad Prism 10 (Graphpad). Data was checked for normality, and the appropriate parametric or non-parametric analysis used accordingly. Details of analyses used are given in figure legends. The level of significance was set at *p* < 0.05. Coefficient of variation (CoV) and effect sizes were additionally calculated for all datasets and are given in Supplementary Table 4. CoV was not significantly different between HC and adult ADHD patient lines, indicating comparable interindividual variability (Supplementary Fig. 3).

## Results

### Optimisation and characterisation of hiPSC-derived cortical neuron model

Our first aim was to develop an hiPSC-based experimental approach to investigate cortical (and thereby glutamatergic) neurons. We were able to successfully generate quality-controlled NPCs and immature cortical neurons (CNs) using small molecule-directed differentiation (Supplementary Fig. 1A) from both HCs and ADHD patients, with the cells displaying cell type-specific morphology (Supplementary Fig. 1B). IF was used for QC (Supplementary Fig. 1C) and confirmed that CN cultures consisted predominantly of glutamatergic neurons (~90%) with a small percentage of astrocytic precursor cells (APCs; ~5%; Supplementary Fig. 1D). Occasional GAD65+ cells were also observed; however, these were rare (Supplementary Fig. 1C). Representative IF images confirming successful neuronal differentiation for all cell lines are given in Supplementary Figs. 5, 6.

**Fig. 1:**
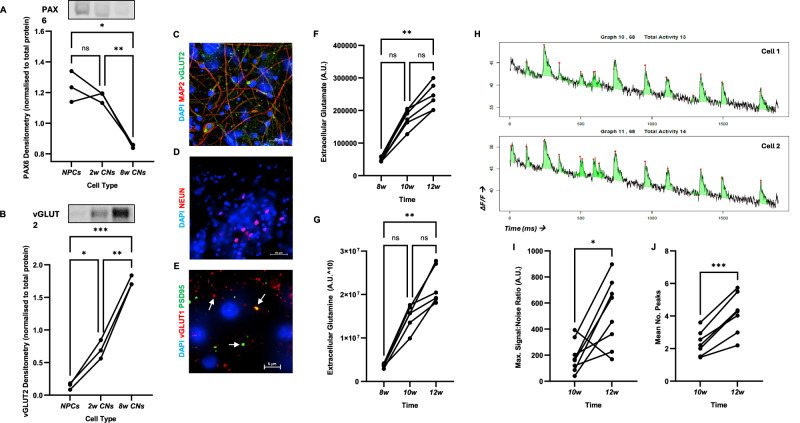
hiPSC-derived CNs from both HCs and ADHD patients could be functionally matured. **A** Western blot showed that PAX6 protein expression significantly decreased during differentiation, consistent with neurogenesis (n = 3). **B** Western blot also showed that vGLUT2 protein expression significantly increased during differentiation, consistent with glutamatergic development (n = 3). **C** IF of 12-week-old CNs showed protein expression of the mature neuron axonal marker MAP2, and vGLUT2, indicating mature glutamatergic neurons. **D** Mature neuronal nuclei marker NEUN was also found expressed. **E** Pre- and post-synaptic markers vGLUT1 and PSD95 (respectively) were also found expressed. Moreover, they were found to colocalise, indicative of glutamatergic synapses. **F** Extracellular glutamate (n = 6) and **G** glutamine (n = 6) were found to significantly increase during neuronal maturation, consistent with the development of functional glutamatergic neurons. **H** Representative calcium imaging traces from two HC neurons showing calcium peaks, confirming that neurons were capable of spontaneous synaptic signalling. **I** Maximum signal:noise ratio (indicative of peak amplitude; n = 8) and **J** mean number of peaks per event (indicative of firing frequency; n = 7) significantly increased with neuronal maturation. Data was analysed using repeated measures one-way ANOVA **A**, **B**, Friedman test **C**, **D** and paired t-test **G**, **H**. NPCs neural progenitor cells, CNs cortical neurons. *p < 0.05, **p < 0.01, ***p < 0.001.

CN cultures from were investigated for their ability to develop into functionally mature cortical neurons capable of spontaneous synaptic signalling. Time-course patterns were constructed for the expression of key proteins involved in cortical neurodevelopment. Protein expression of PAX6 was found to significantly decrease during neuronal differentiation from NPCs to 8-week-old CNs (*p* = 0.0151, Fig. 1A), whereas vGLUT2 protein expression was found to significantly increase during this time-period (*p* = 0.0001, Fig. 1B). IF labelling of 12-week-old CNs showed protein expression of the mature neuron somatodendritic marker MAP2, as well as the glutamatergic protein vGLUT2 (Fig. 1C), suggesting the presence of mature glutamatergic neurons. Representative IF images for each cell line are shown in Supplementary Fig. 7. CNs also expressed TUBB3 and the mature neuron nuclear marker NEUN (Fig. 1D). Lastly, 12-week-old CNs were found to express both the pre- and post-glutamatergic synapse markers vGLUT1 and PSD95 (respectively; Fig. 1E). Moreover, these markers were also found colocalised, suggesting the presence of glutamatergic synapses (singular and colocalised markers are shown by white arrows). For 3D rendering see Supplementary Fig. 8.

Next, the production of extracellular glutamate (Fig. 1F) and glutamine (Fig. 1G) was assessed and found to significantly increase during neuronal maturation (both *p* = 0.0001). Lastly, live calcium imaging was performed to confirm synaptic signalling. Figure 1H shows calcium signal traces from two healthy control (HC) neurons (fluorescence over time), with calcium peaks depicted by red dots, indicative of spontaneous calcium signalling. Quantification of calcium signals revealed a significant increase in maximum signal:noise ratio (*p* = 0.0145, Fig. 1I) and mean number of calcium peaks over time (*p* = 0.0003, Fig. 1J), suggesting increase signalling strength and frequency with neuronal maturation.

### Differentiation capacity of hiPSCs derived from HCs and ADHD patients

**Fig. 2:**
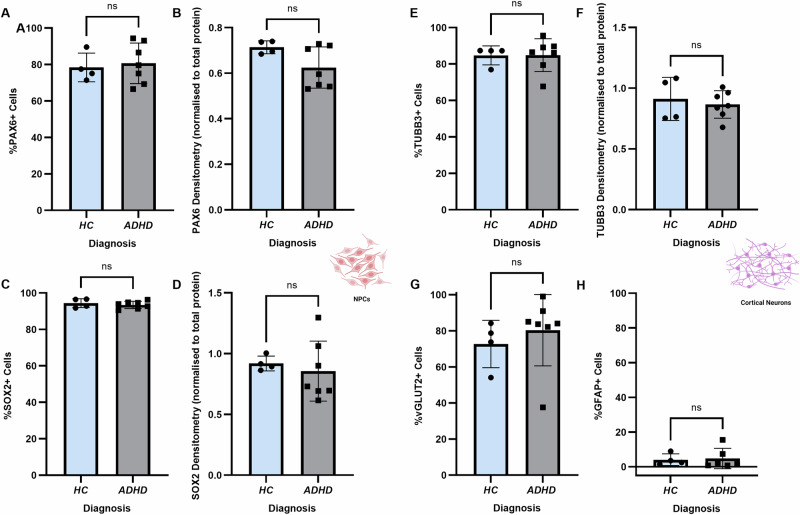
hiPSCs derived from HC and ADHD patients do not differ in their ability to undergo neuronal differentiation. **A** IF showed that hiPSC-derived NPCs from HC (n = 4) and ADHD patients (n = 7) showed similar percentages of cells expressing PAX6, and **B** there were no changes in overall PAX6 protein expression. **C** hiPSC-derived NPCs from HC and ADHD patients showed similar percentages of cells expressing SOX2 and **D** overall SOX2 protein expression did not differ. **E** IF of 2-week-old CNs showed that HC (n = 4) and ADHD patient cultures (n = 7) had similar cell proportions expressing TUBB3, and **F** there were no changes in overall TUBB3 protein expression. There were also no differences in the percentage of cells expressing **G** vGLUT2, nor **H** GFAP. NPCs neural progenitor cells, CNs cortical neurons.

Once we had established the model, we next aimed to use it to investigate the pathomechanisms underlying ADHD. We firstly assessed whether hiPSCs derived from HC and adult ADHD patients differed in their abilities to form NPCs and CNs. There were no differences in the percentage of PAX6 expressing cells in NPC cultures from HC and ADHD patients (Fig. 2A), nor in the overall amount of PAX6 protein expressed (Fig. 2B). The same results were observed for SOX2 (Fig. 2C, D). Upon differentiation into immature (2-week-old) CNs, there were no differences in the number of neurons (TUBB3+ cells) present between HC and ADHD patients (Fig. 2E), nor were there any differences in the overall protein expression of TUBB3 (Fig. 2F). Lastly, no differences in the proportion of glutamatergic neurons (vGLUT2+; Fig. 2G) or APCs (GFAP+; Fig. 2H) present were observed, suggesting that hiPSCs from ADHD patients were able to undergo neuronal differentiation with a similar efficiency to HCs.

**Fig. 3:**
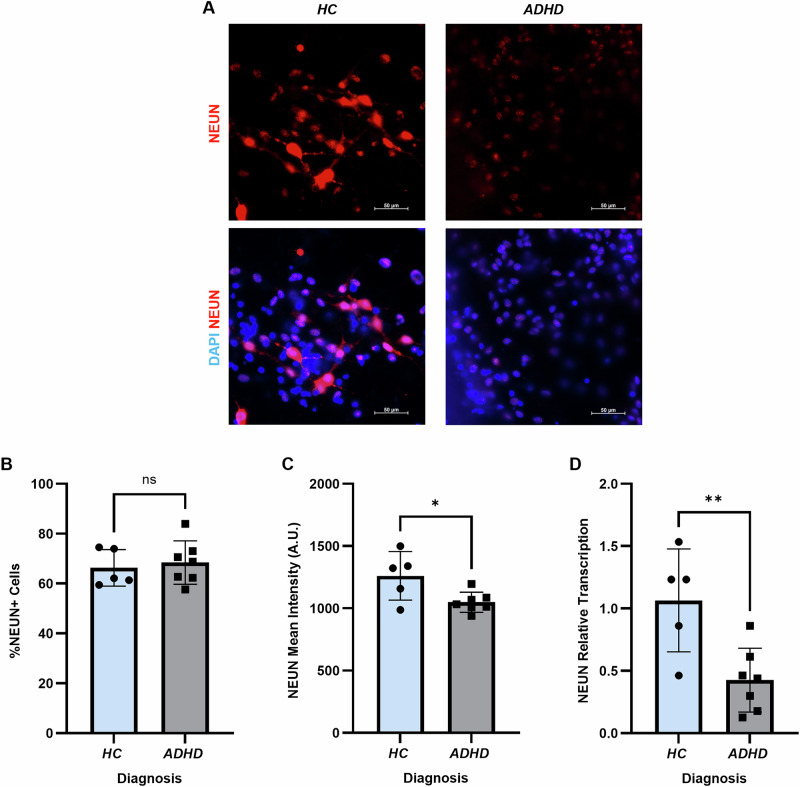
Inhibited maturation of ADHD patient hiPSC-derived CNs. **A** IF of 12-week-old CNs showed NEUN+ positive nuclei in both HC and ADHD patient cultures, however labelling intensity appeared decreased in ADHD patient CNs. LUTs were adjusted to the same values to enable comparability. **B** Quantification of nuclei expressing NEUN showed no differences between HC and ADHD patient CNs. **C** Quantification of NEUN labelling intensity showed a significant decrease in ADHD patient CNs. **D** RT-qPCR showed significantly decreased NEUN transcription in ADHD patient CNs. All data was analysed using independent t-tests. CNs cortical neurons, HC healthy controls. *p < 0.05. **p < 0.01.

We next wanted to investigate whether there were changes in neuronal maturation in ADHD patient CNs. After maturation into 12-week-old functional neurons, IF labelling for NEUN was performed (Fig. 3A). Quantification showed that there was no significant difference in the number of nuclei positive for NEUN (Fig. 3B). However, there was a significant decrease in the fluorescent intensity of NEUN in CNs from ADHD patients, suggesting reduced protein expression (*p* = 0.0245; Fig. 3C). To confirm this finding, we then performed RT-qPCR on 12-week-old CNs, which also revealed a significant decrease in NEUN transcription in ADHD patients (*p* = 0.0076; Fig. 3D).

### Differences in glutamatergic development

**Fig. 4:**
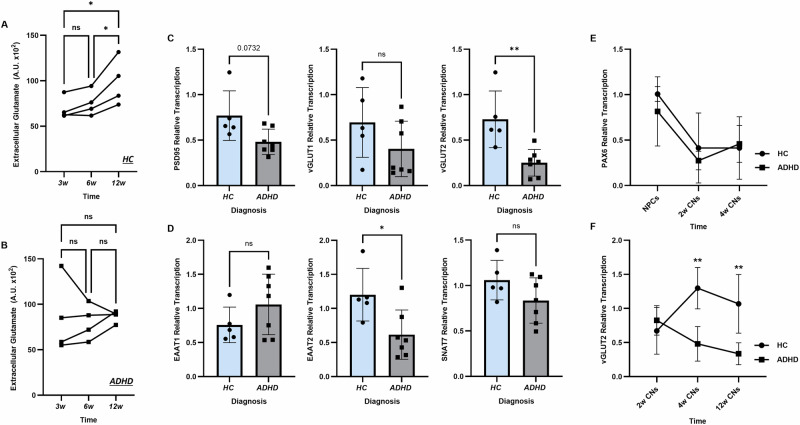
hiPSC-derived CNs from ADHD patients show altered glutamatergic development. **A** Extracellular glutamate assay revealed a significant increase in concentration in HC CNs during maturation (n = 4). **B** No significant increase in extracellular glutamate was observed in ADHD patient CNs (n = 4). **C** Expression of glutamatergic signalling genes was investigated in mature 12-week-old CNs from HC (n = 5) and ADHD patients (n = 7) using RT-qPCR. *vGLUT2* transcription was found significantly decreased in ADHD patient CNs by ~50%. **D** Expression of glutamate transport genes was also investigated. No significant differences in *EAAT1* or *SNAT7* transcription were found. However, a significant reduction in *EAAT2* transcription was found in ADHD patient CNs. To assess potentially altered neurodevelopment, **E** *PAX6* and **F** *vGLUT2* transcription was determined over time in HC (n = 4) and ADHD patients (n = 4). There were no significant differences in *PAX6* transcription over time. However, *vGLUT2* transcription showed a distinct course, with significantly reduced *vGLUT2* gene expression found at 4 and 12 weeks. Data was analysed using repeated measures one-way ANOVA **A**, **B**, independent t-tests **C**, **D** and two-way ANOVA (**E**, **F**). CNs cortical neurons, HC healthy controls. *p < 0.05, **p < 0.01.

We next set out to investigate glutamatergic development, starting with changes in extracellular glutamate concentration during differentiation. In HC CNs, there was a significant increase in extracellular glutamate concentration over time, consistent with the release of glutamate from glutamatergic neurons (*p* = 0.0276, Fig. 4A). However, this pattern was not found in CNs from ADHD patients; extracellular glutamate concentrations appeared to remain similar across development (Fig. 4B). We next investigated the expression of several genes involved in glutamatergic signalling using 12-week-old CNs, as our characterisation had shown the neurons were functionally mature at this time point. Mean relative *PSD95* transcription in CNs from healthy controls was found to be 0.66, and 0.44 in ADHD patients, and mean relative *vGLUT1* transcription was found to be 0.69 and 0.4 respectively (Fig. 4c). However, neither of these results were statistically significant (*p* = 0.0732 and *p* = 0.1727, respectively). We found a significant decrease in *vGLUT2* transcription, which was approximately 50% lower in ADHD patient CNs (*p* = 0.005). We next looked at gene expression of glutamate transporters specifically selected for their previously reported associations with neuropsychiatric disorders; *EAAT1* (ADHD [36]), *EAAT2* (bipolar disorder, schizophrenia [37]) and *SNAT7* (schizophrenia [38]). No significant differences in transcription were observed for *EAAT1* (Fig. 4D) or *SNAT7*. However, a significant decrease in *EAAT2* transcription was found in CNs from ADHD patients (*p* = 0.0227).

Based on our findings of altered developmental pattern and glutamatergic gene expression, we next determined the transcription of *PAX6* and *vGLUT2* over time, to assess neurogenesis and glutamatergic development respectively. *PAX6* transcription over time appeared to follow a similar pattern in both HC and ADHD patient CNs, with transcription decreasing during neuronal differentiation (Fig. 4E). For *vGLUT2*, there were no significant differences in transcription in immature, 2-week-old neurons (Fig. 4F). However, as development progressed, the course of *GLUT2* gene expression diverged. HC CNs displayed a distinct upregulation of *vGLUT2* transcription with time, whereas *vGLUT2* transcription in ADHD patient CNs decreased with time, with significantly lower transcription observed at both 4-week and 12-week time points (*p* = 0.0088). Graphs showing individual data points are provided in Supplementary Fig. 10.

### Functional effects on glutamatergic synaptic signalling

**Fig. 5:**
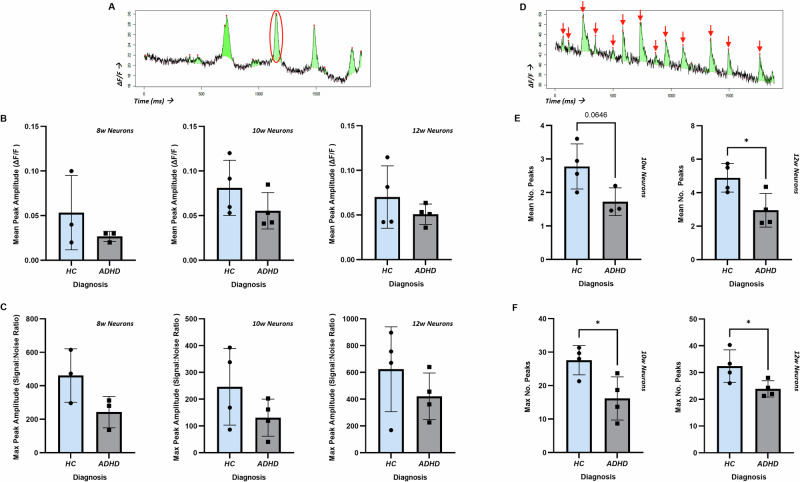
Calcium imaging indicates a hypoactive phenotype in ADHD patient CNs. **A** Representative calcium imaging trace of one HC neuron depicting peak amplitude (red circle). **B** Mean peak amplitude was measured at 8, 10 and 12 weeks during neuronal maturation in HC (n = 4; except for 8-week measurement, n = 3) and ADHD patients (n = 4; except for 8-week measurement, n = 3), and a trend for decreased mean peak amplitude in ADHD patient CNs was observed at all time points. **C** The same trend was observed when assessing maximum peak amplitude in the same cells. **D** Representative calcium imaging trace of one HC neuron depicting calcium peaks (red circle). **E** Mean number of calcium peaks over time was quantified at 10 and 12 weeks in HC (n = 4) and ADHD patient (n = 4) CNs. A marginally significant decrease in calcium peak rate was observed at 10 weeks, and a significant decrease at 12 weeks. **F** Maximum calcium peak rate reflected these results, with significant decreases observed at both 10 and 12 weeks. Data was analysed using independent t-tests. CNs cortical neurons, HC healthy controls. *p < 0.05.

Given our findings of altered glutamatergic development, we lastly investigated whether glutamatergic signalling was impacted as a functional consequence. We again investigated the developmental course over time, using live calcium imaging of functionally mature CNs. We firstly assessed the peak amplitude of calcium signals, as shown in a representative trace from one HC neuron (Fig. 5A). No significant differences were found, however there was a clear trend for decreased mean (Fig. 5B) and maximum (Fig. 5C) peak amplitude in ADHD patient CNs at all time points analysed. We lastly investigated the number of calcium peaks in a given time period as a measure of neuronal activity, as demonstrated in a representative calcium trace from one HC neuron (calcium peaks denoted by red arrows; Fig. 5D). We found significantly decreased mean calcium peak rates in CNs from ADHD patients, which were stable over time (10-week and 12-week-old CNs, *p* = 0.0646 and *p* = 0.0256 respectively, Fig. 5E). Significantly decreased maximum calcium peak rates were also observed (10-week and 12-week-old CNs, *p* = 0.0264 and *p* = 0.0458 respectively, Fig. 5F).

## Discussion

We were able to establish an hiPSC-derived functionally mature CN model for the investigation of ADHD pathomechanisms. The small molecule-based model involved the generation of intermediate NPCs expressing PAX6 and SOX2, followed by differentiation into CNs consisting of predominantly glutamatergic neurons. Moreover, these CNs could be matured for several weeks, forming glutamatergic synapses and capable of spontaneous signalling.

We found that there were no changes in the cell proportions generated from healthy control donors and ADHD patients, suggesting both were able to successfully differentiate into CNs. We additionally investigated whether hiPSCs from both groups could form mature neurons and found a similar percentage of NEUN+ nuclei in the cultures. However, we observed a significant decrease in NEUN fluorescent intensity in ADHD patient CNs, potentially indicating decreased protein expression. As IF is only semi-quantitative and requires careful calibration and optimisation, this finding requires further validation using more sensitive methods, such as western blot. Decreased NEUN protein expression was however supported by RT-qPCR in our study, which revealed significantly decreased *NEUN* transcription in ADHD patient CNs. Previously, dysfunctional NEUN has been implicated in epilepsy [39], cognitive impairments [40], neurodevelopmental delay [41] and autism spectrum disorder (ASD) [42], but not ADHD. However, ADHD is the most common comorbidity in children with epilepsy [43], and ~28% individuals with ASD also have comorbid ADHD [44]. Moreover, cognitive impairments and neurodevelopmental delay are phenotypes strongly associated with ADHD [17, 45]. Our results are also consistent with the brain maturation delay theory of ADHD [17], and suggest that NEUN may play a previously unidentified role in ADHD pathology.

We further centred our study on investigation of the glutamatergic system, to determine whether we could identify changes in ADHD patient CNs. As part of quality control, we quantified extracellular glutamate, and observed that CNs from ADHD patients had a different pattern to healthy controls. Significant increases in extracellular glutamate were found in healthy controls, but no significant increases were observed in ADHD patients. These results are consistent with previous studies in humans using spectroscopic methods, with a recent meta-analysis revealing significantly altered glutamate-glutamine concentrations in the medial PFC of ADHD patients compared to healthy controls [46]. However, this finding should be interpreted with caution, as changes in extracellular glutamate over time were variable in hiPSC-derived cortical neurons from ADHD patients, with only two out of four donors not demonstrating the expected increase as shown in the healthy control donors. Future studies need to further investigate whether glutamate synthesis is truly affected in ADHD, including a higher number of donors to account for variability. For example, metabolomic analysis could be performed to determine intra- and extracellular glutamate and glutamine concentrations.

Our results also revealed changes in the expression of genes involved in glutamatergic signalling and transport. Firstly, *PSD95* transcription was found to be lower in ADHD patient CNs, although it should be noted that this result did not reach significance. In recent years, a new rare brain disorder was identified termed ´DLG4-related synaptopathy´ [47]. This disorder is characterised by genetic variants in *PSD95* causing deficient protein expression, resulting in clinical features such as developmental delay and epilepsy. Moreover, one of the main associated clinical presentations was ADHD. We additionally found significantly decreased transcription of *EAAT2* in ADHD patient CNs, which is responsible for up to ~95% glutamate uptake in the CNS [48], correlating with our finding of altered extracellular glutamate. *EAAT2* has additionally previously been associated with bipolar disorder and schizophrenia [37], both of which are commonly found comorbid with ADHD [49, 50]. Although primarily expressed in astrocytes [51], it has been proposed that *EAAT2* may also be expressed in neurons [52], particularly during cortical development [53]. It is therefore unclear whether the decreased transcription we observed may be due to neuronal or astrocytic *EEAT2* gene expression and needs to be further clarified.

Our most significant finding was an ~50% decrease in *vGLUT2* transcription in CNs from ADHD patients compared to healthy controls. To further investigate this, we additionally investigated the course of *vGLUT2* transcription during differentiation and found divergent paths after 2 weeks, with consistent inhibited transcription in ADHD patients. The data therefore suggests that decreased *vGLUT2* gene expression is a consistent and robust cellular disease endophenotype. To the authors’ knowledge, this is the first evidence for a direct role of *vGLUT2* in ADHD pathogenesis. There is very little in the literature regarding *vGLUT2* and neuropsychiatric disorders in general. One previous paper investigating the effects of reduced *Vglut2* gene expression in subthalamic nuclei in mice observed behavioural hyperlocomotion and reduced postsynaptic activity as a consequence [54]. Another study in mice focusing on the cognitive effects of cisplatin reported decreased *Vglut2* in the cortex of treated mice, which was specifically associated with attention deficits [55]. Lastly, a single nucleotide polymorphism in the *vGLUT2* gene was found to moderate environmental sensitivity to alcohol-related problems in youth [56], and it has been well-documented that substance use disorders have a high comorbidity with ADHD [57]. It also possible that the *vGLUT2* gene is not directly involved in the heritability of ADHD, and instead represents a downstream core process through which the functional impact of multiple genetic aberrations converges, as recently proposed for complex traits and polygenic disorders [58].

To investigate whether changes in gene expression could affect neuronal functioning, we performed calcium imaging to assess synaptic signalling. We found that there was a consistent trend for decrease peak amplitude in ADHD patient CNs, inferring reduced signalling strength. We additionally observed a significant decrease in calcium peak rates. Together, this suggests a hypoactive phenotype in CNs from ADHD patients. A previous study using the spontaneously hypertensive rat model of ADHD also found evidence of disrupted glutamatergic neurotransmission in the PFC, reporting significantly decreased AMPAR-mediated synaptic transmission [59]. Moreover, a functional near-infrared spectroscopy study additionally found significantly decreased activation of the PFC in children with ADHD when completing the Go/No-Go task [60], further suggesting a hypoexcitable glutamatergic phenotype in ADHD pathology.

Taken together, our results provide a potential functional disease mechanism in ADHD pathology. Inhibited neuronal maturation and decreased *vGLUT2* transcription could result in reduced glutamate release at the synaptic cleft, resulting in imbalanced extracellular glutamate concentrations and decreased gene expression of the primary glutamate uptake transporter *EAAT2*. Moreover, inhibited glutamate release could be causal for the decreased synaptic signalling observed. However, to determine whether altered glutamate synthesis, delayed neuronal development or both be responsible for results obtained in this preliminary study, further studies specifically investigating these mechanisms are needed. Now that we have established a potential cellular disease phenotype, our model could be suitable for drug screening in order to identify compounds capable of reversing this phenotype. Several effective ADHD medications, including MPH and atomoxetine, have previously been shown to have glutamatergic effects [61]. Moreover, several medications specifically targeting the glutamatergic system have already been used in human clinical trials with improvements observed [61].

There are several limitations to this study. Firstly, 2D neuronal cultures were used, which lack spatiotemporal cues and the complexity of an in vivo setting (e.g. less cell types, no vascularisation) [25]. Secondly, although our sample size adhered to recent recommendations [62], it is still comparatively small and should be expanded to increase the power to detect more modest expressional and functional changes. Moreover, hiPSC models are known to show high interindividual variability, which was also the case in this study [63]. We attempted to quantify this variability by calculating the coefficient of variation, and did not find any significant differences between healthy controls and adult ADHD patient lines. Thirdly, as heterogeneous cortical cultures were used consisting of both neurons and astrocytes, it is possible that cell type-specific alterations with small effect sizes were not identified. Lastly, there was a small male bias in ADHD donors, reflecting the clinical diagnostic bias [11].

In summary, we established a functional cortical neuronal model to investigate the potential role of the glutamatergic system in ADHD pathology. Using this model, we were able identify altered developmental paths and dysregulated expression of genes involved in neuronal maturation, glutamatergic synapses and glutamate transport. Reduced *vGLUT2* gene expression was identified as a robust cellular disease endophenotype, along with neuronal hypoactivity, which could potentially be used for future drug screening. Further studies should seek to replicate these findings in more complex settings (e.g. hiPSC-derived cortical organoids) and explore other potential functional consequences of altered glutamatergic development in ADHD patients (e.g. glutamatergic synapse development).

## Supplementary information

Supplementary Materials

## Data Availability

Supplementary information is available at MP’s website. Raw data is available from the corresponding author.
