# Supplementary material for: hiPSC-derived cortical neurons from ADHD individuals reveal dysregulated glutamatergic development
Source: Mol Psychiatry. 2025 Sep 19;31(2):1041–50. doi: 10.1038/s41380-025-03213-8 (PMC12815685; doi:10.1038/s41380-025-03213-8)
Supplement: Supplementary file 1 — Supplementary Materials [file 41380_2025_3213_MOESM1_ESM.pdf]

# Supplementary Information

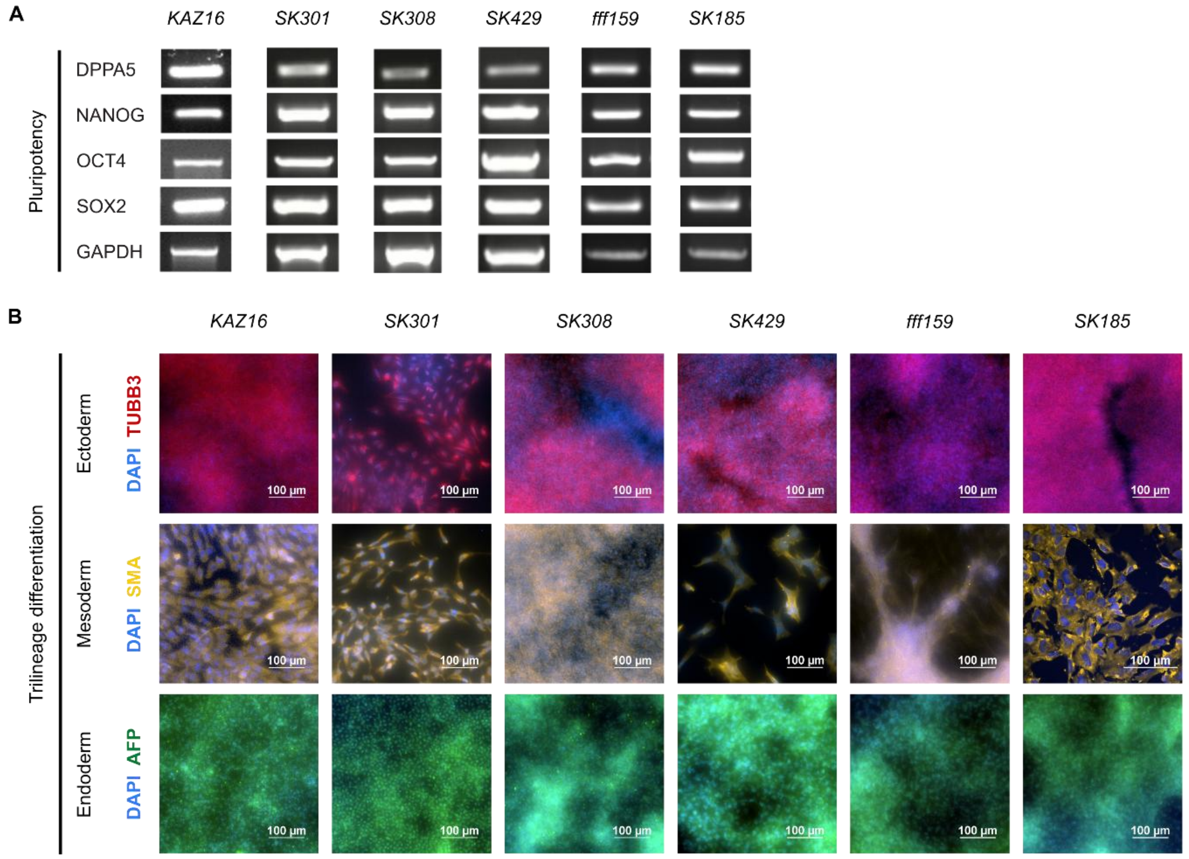


**Supplementary Figure 1. Characterisation of hiPSCs.** All hiPSC lines used in this study were quality controlled as described in the Materials and Methods. For hiPSC lines previously not published, basic QC is provided. (A) RT-PCR for pluripotency-associated genes and the housekeeping gene *GAPDH*. (B) hiPSCs were differentiated into the three germ layers using small molecules and IF performed for the following protein markers: TUBB3 (ectoderm), SMA (mesoderm) and AFP (endoderm).

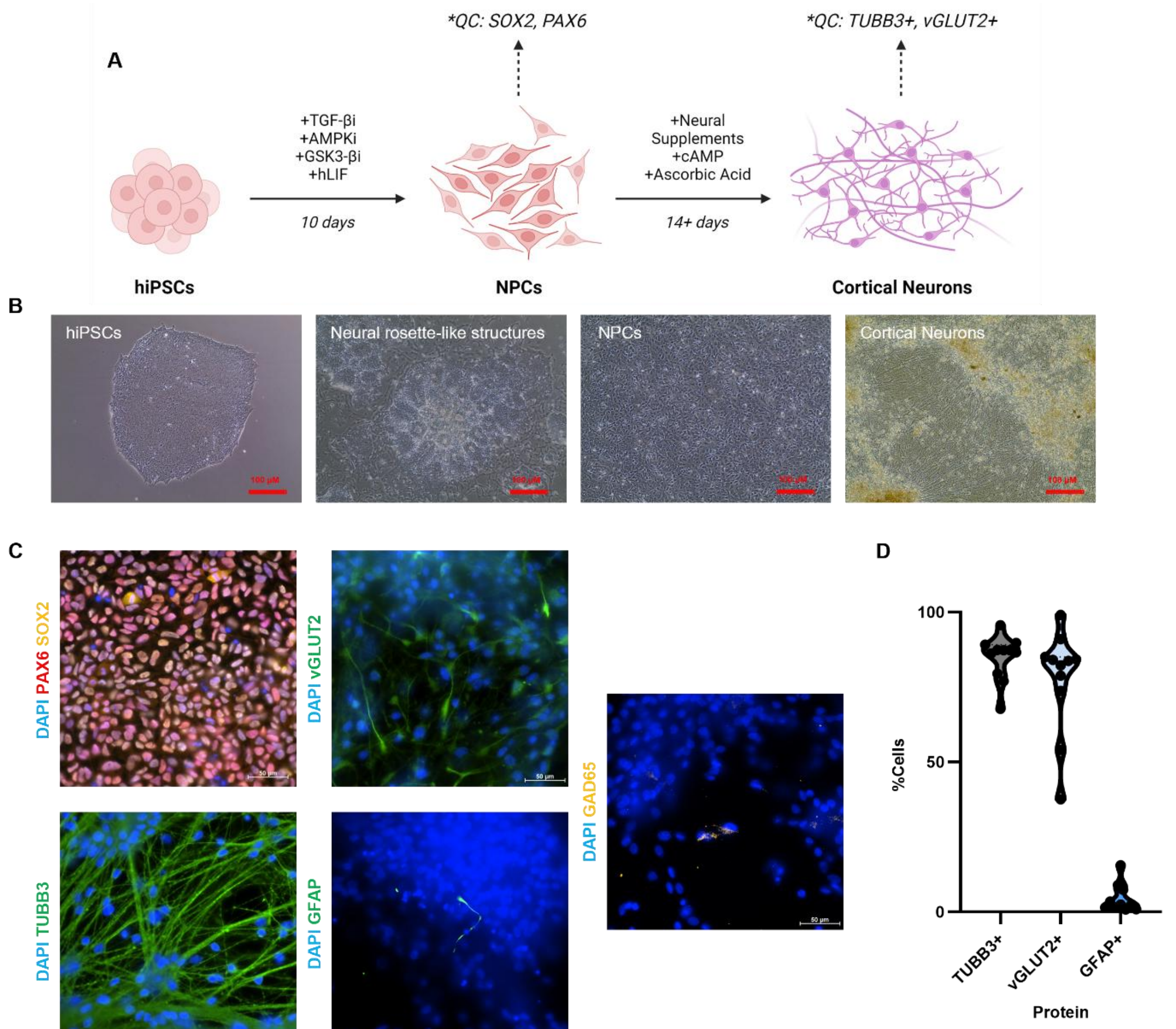


**Supplementary Figure 2. Establishment and characterisation of hiPSC-derived NPCs and CNs.** (A) Workflow for generating hiPSC-derived NPCs and CNs, including quality control steps. Created in BioRender. Kittel-schneider, S. (2024) BioRender.com/o42s763). (B) Representative phase contrast images of different cell types throughout differentiation. NPCs showed a polarised morphology and CNs showed extensive axonal outgrowth. (C) Representative IF images of quality control performed on resultant NPCs and CNs. ≥75 % NPCs were required to express SOX2/PAX6 to be used for further experiments. CNs expressed TUBB3 and vGLUT2, and a small number of cells expressed GFAP. Occasionally, GAD65+ cells were observed. (D) Cell proportions in 14-day-old CN cultures (n=11). Most cells expressed TUBB3+vGLUT2,

indicating glutamatergic neurons. A small percentage of cells expressed GFAP, indicative of astrocytic precursors. NPCs=neural progenitor cells, CNs=cortical neurons.

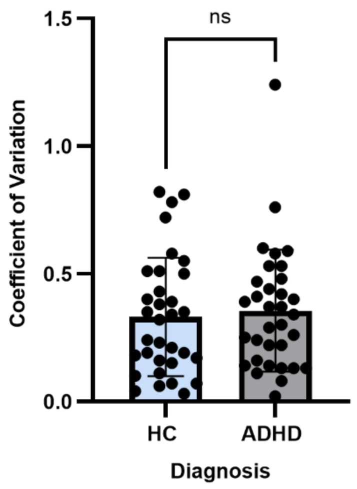


**Supplementary Figure 3. Coefficient of variance analysis for all datasets.** Data variance was quantified by calculating the coefficient of variation (CoV) for each experimental dataset. No significant increase in CoV was found between healthy controls and ADHD patients.

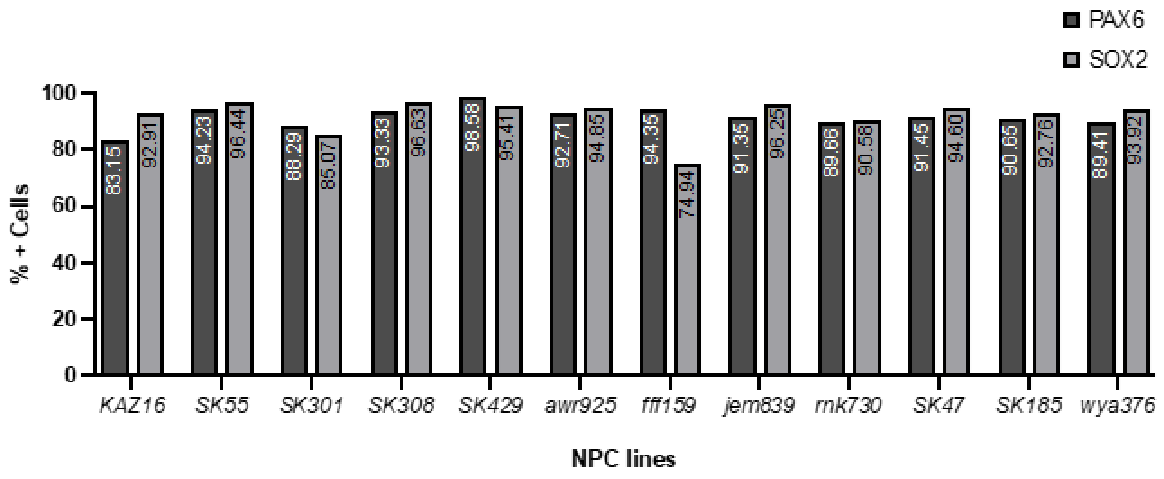


**Supplementary Figure 4. Quality control of hiPSC-derived NPCs.** The protein expression of the NPC-associated markers SOX2 and PAX6 was confirmed using IF. NPCs were only used if ≥75% cells expressed these markers. NPCs=neural progenitor cells.

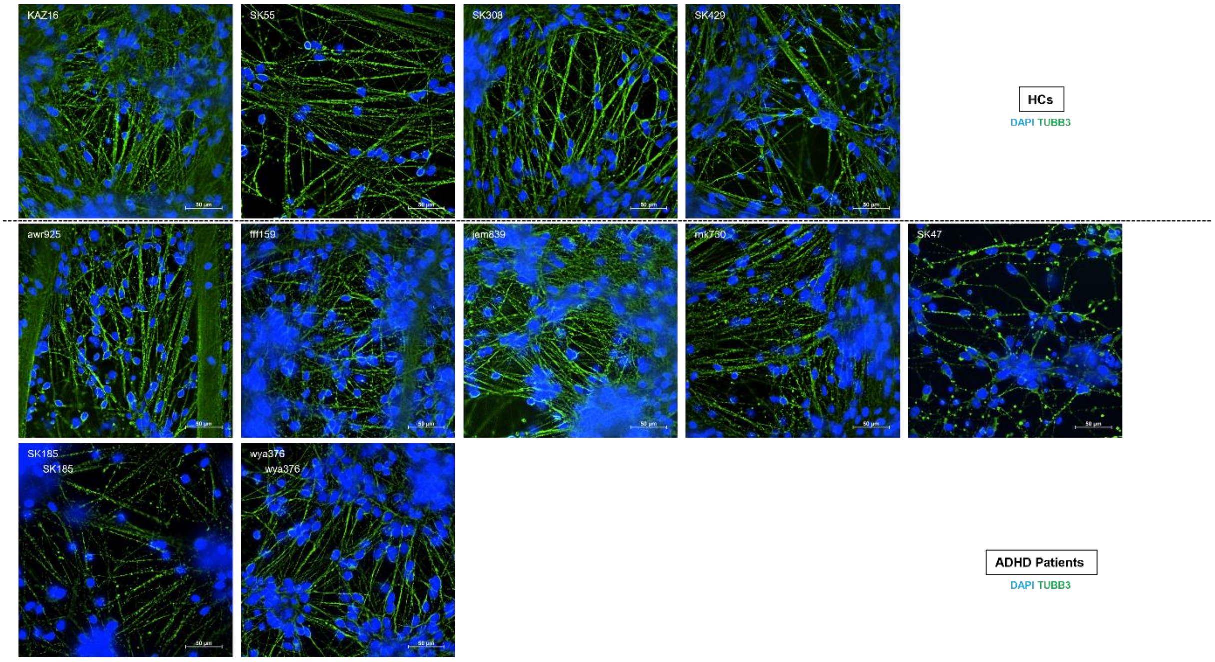


**Supplementary Figure 5. Representative IF images for each cell line showing TUBB3 expression in 2-week-old cortical neurons.** IF was performed for TUBB3 protein expression for every cell line to confirm successful neuronal differentiation.

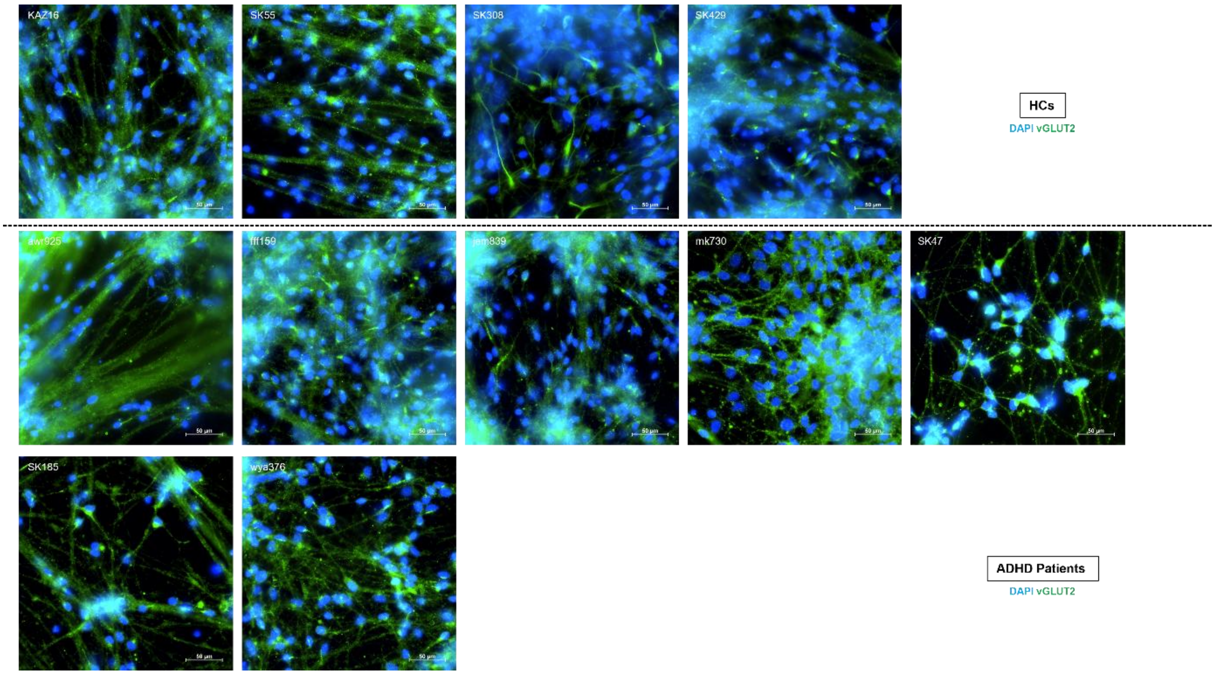


**Supplementary Figure 6. Representative IF images for each cell line showing vGLUT2 expression in 2-week-old cortical neurons.** IF was performed for vGLUT2 protein expression for every cell line to confirm successful glutamatergic neuron differentiation.

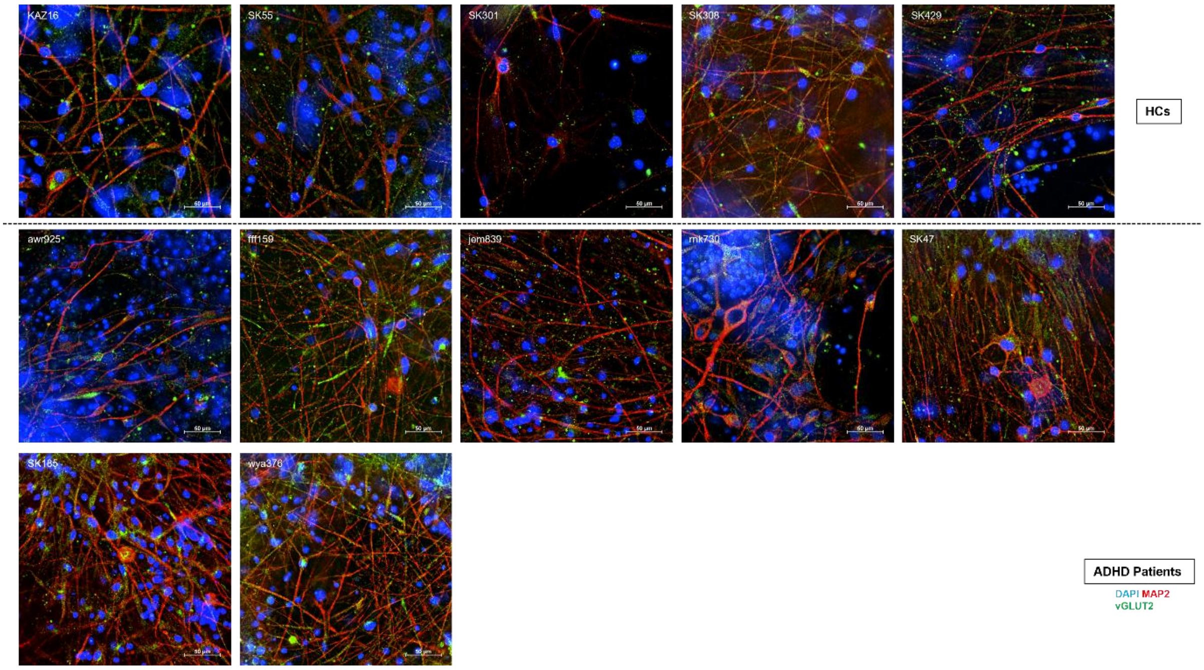


**Supplementary Figure 7. Representative IF images for each cell line showing MAP2 and vGLUT2 expression in 12-week-old cortical neurons.** IF was performed for MAP2 and vGLUT2 protein expression for every cell line to confirm successful mature glutamatergic neuron differentiation.

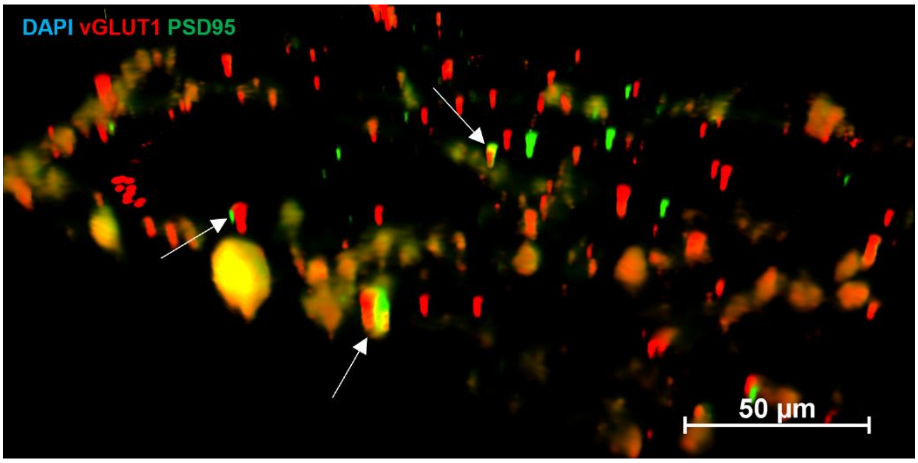


**Supplementary Figure 8. Representative 3D rendering glutamatergic synapses.** IF was performed for vGLUT1 and PSD95 protein expression and confocal microscopy performed to confirm the presence of glutamatergic synapses. 3D rendering of Z-stack images showed colocalisation of vGLUT1 and PSD95 proteins, suggesting the presence of glutamatergic synapses.

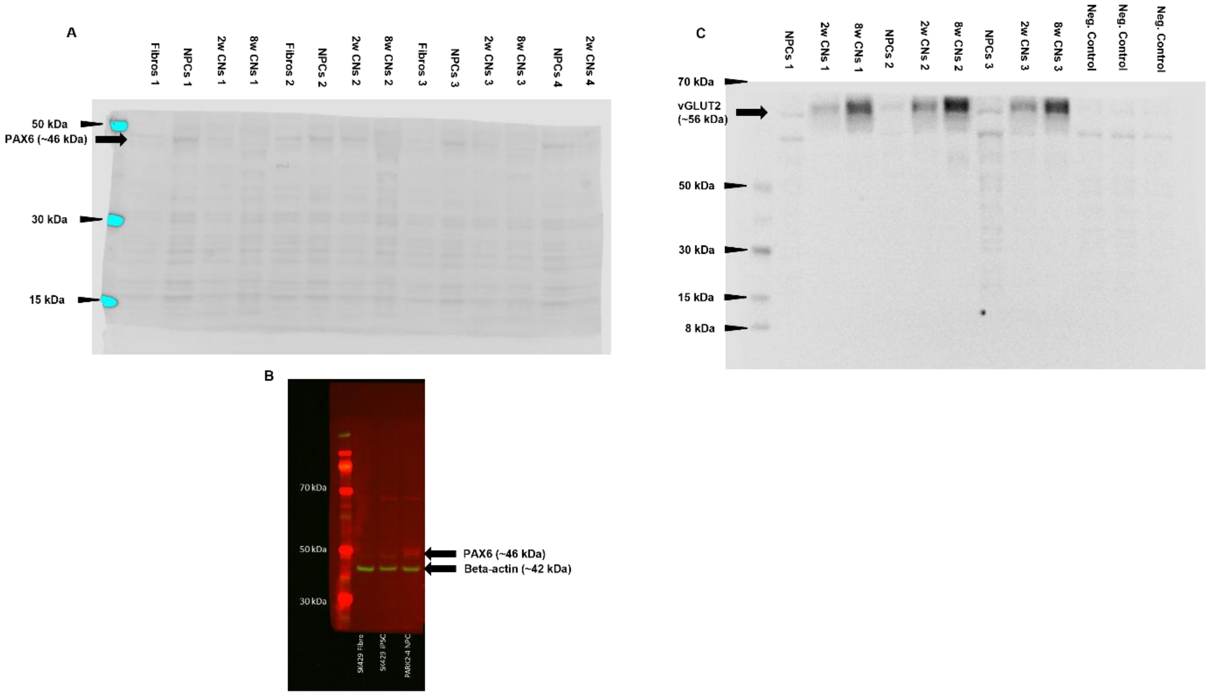


**Supplementary Figure 9. Uncropped western blot images for detecting PAX6 and vGLUT2 protein expression.** (A) PAX6 band is visible at the correct molecular weight of 46 kDa, predominantly in NPC protein lysates. (B) PAX6 band is visible only in NPC protein lysates, and not fibroblast cells or hiPSCs. (C) vGLUT2 band is visible at the correct molecular weight of 56 kDa, with highest expression observed in 8-week-old cortical neurons, and no bands observed in negative controls (fibroblast cells). Fibros=fibroblast cells, NPCs=neural progenitor cells, CNs=cortical neurons.

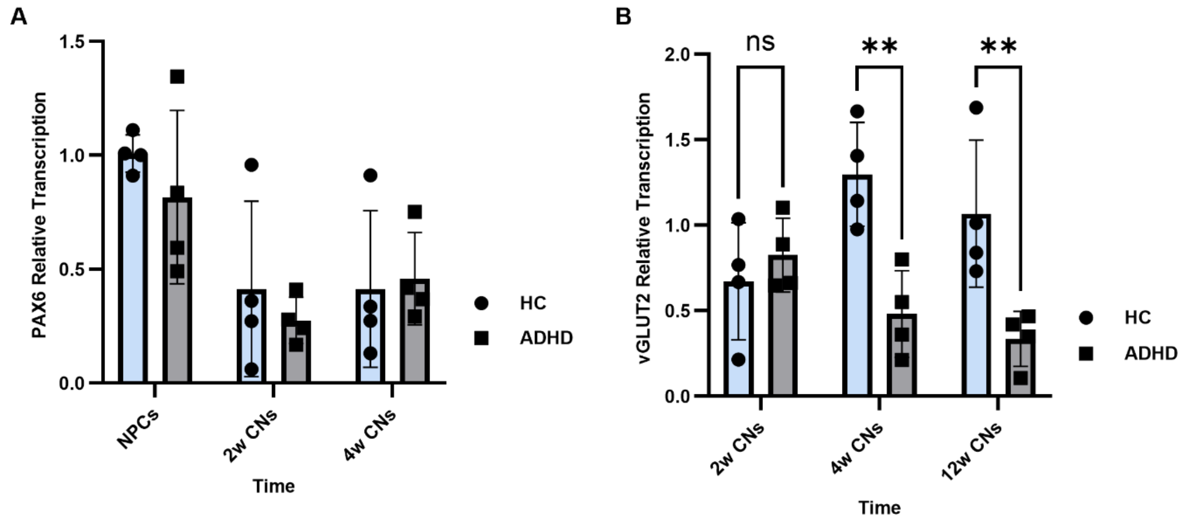


**Supplementary Figure 10. PAX6 and vGLUT2 relative transcription across glutamatergic development with individual data points shown.** (A) PAX6 relative transcription and (B) vGLUT2 relative transcription. NPCs=neural progenitor cells, CNs=cortical neurons.

**Supplementary Table 1.** Details of donors from which hiPSC lines were generated. hiPSCs that have previously been published are denoted with their references. All hiPSC line were quality controlled as described in the methods.

| Donor Code | Age | Sex | Diagnosis | Reference |
|---|---|---|---|---|
| KAZ16 | 45 | Male | Healthy | N/A |
| SK55 | 42 | Female | Healthy | [25, 26] |
| SK301 | 25 | Male | Healthy | N/A |
| SK308 | 28 | Male | Healthy | N/A |
| SK429 | 27 | Female | Healthy | [25, 26] |
| awr925 | 27 | Male | ADHD | [32] |
| fff159 | 58 | Female | ADHD | N/A |
| jem839 | 39 | Male | ADHD | [32] |
| rnk730 | 46 | Male | ADHD | [32] |
| SK47 | 28 | Female | ADHD | N/A |
| SK185 | 53 | Male | ADHD | N/A |
| wya376 | 39 | Male | ADHD | [32] |

**Supplementary Table 2.** Taqman™ primer assays used for RT-qPCR.

| Target Gene | Assay ID |
|---|---|
| PAX6 | Hs01088114_m1 |
| vGLUT2/SLC17A6 | Hs00220439_m1 |
| vGLUT1/SLC17A7 | Hs00220404_m1 |
| PSD95/DLG4 | Hs01555373_m1 |
| EAAT1/SLC1A3 | Hs00904823_g1 |
| EAAT2/SLC1A2 | Hs01102423_m1 |
| SNAT7/SLC38A7 | Hs01046689_m1 |
| SDHA | Hs07291714_m1 |
| GUSB | Hs00939627_m1 |
| HMBS | Hs00609296_g1 |
| TBP | Hs00427620_m1 |
| GAPDH | Hs02786624_g1 |
| YWHAZ | Hs01122445_g1 |

**Supplementary Table 3.** Primary and secondary antibodies used.

| Antibody | Cat# | Supplier | Dilution |
|---|---|---|---|
| Anti-PAX6 | PRB-278P | BioLegend | IF 1 in 500; WB 1 in 1500 |
| Anti-SOX2 | 3579T | Cell Signalling | IF 1 in 500; WB 1 in 1000 |
| Anti-TUBB3 | 66375-1-Ig | ProteinTech | IF 1 in 500 |
| Anti-vGLUT2 | MAB5504 | Merck | IF 1 in 500; WB 1 in 3000 |
| Anti-GFAP | MAB3402 | Merck | IF 1 in 2000 |
| Anti-GAD65 | MAB351R | Merck | IF 1 in 1000 |
| Anti-MAP2 | 17490-1-AP | ProteinTech | IF 1 in 1000 |
| Anti-NEUN | 26975-1-AP | ProteinTech | IF 1 in 250 |
| Anti-vGLUT1 | 48-2400 | Invitrogen | IF 1 in 250 |
| Anti-PSD95 | MA-046 | Invitrogen | IF 1 in 250 |
| Alexa Fluor 488 goat anti-mouse IgG1 | AB_2651011 | ThermoFisher | 1 in 500 |
| Alexa Fluor 647 donkey anti-rabbit IgG | AB_2651010 | ThermoFisher | 1 in 500 |
| Alexa Fluor 555 goat anti-mouse IgG2a | AB_2651012 | ThermoFisher | 1 in 500 |

**Supplementary Table 4.** Coefficient of variation (CoV) and effect size (Cohen's *d*) were calculated for all experiment datasets.

| Figure | Dataset | Groups | CoV | Cohen's *d* |
|---|---|---|---|---|
| 1F | Extracellular glutamate | 8w | 0.14 | / |
| | | 10w | 0.16 | / |
| | | 12w | 0.16 | / |
| 1G | Extracellular glutamine | 8w | 0.11 | / |
| | | 10w | 0.19 | / |
| | | 12w | 0.20 | / |
| 1I | Calcium Max Signal:Noise Ratio | 10w | 0.64 | / |
| | | 12w | 0.50 | / |
| 1J | Calcium Mean No. Peaks | 10w | 0.31 | / |
| | | 12w | 0.28 | / |
| 2A | % PAX6+ Cells | HC | 0.10 | / |
| | | ADHD | 0.14 | / |
| 2B | PAX6 Densitometry | HC | 0.04 | / |
| | | ADHD | 0.14 | / |
| 2C | %SOX2+ Cells | HC | 0.03 | / |
| | | ADHD | 0.02 | / |
| 2D | SOX2 Densitometry | HC | 0.07 | / |
| | | ADHD | 0.16 | / |
| 2E | %TUBB3* Cells | HC | 0.06 | / |
| | | ADHD | 0.11 | / |
| 2F | TUBB3 Densitometry | HC | 0.19 | / |
| | | ADHD | 0.13 | / |
| 2G | %vGLUT2+ Cells | HC | 0.18 | / |
| | | ADHD | 0.25 | / |
| 2H | %GFAP+ Cells | HC | 0.82 | / |
| | | ADHD | 1.24 | / |
| 3B | %NEUN+ Cells | HC | 0.11 | / |
| | | ADHD | 0.13 | / |
| 3C | NEUN Mean Intensity | HC | 0.15 | / |
| | | ADHD | 0.08 | *1.42* |
| 3D | NEUN Relative Transcription | HC | 0.39 | |
| | | ADHD | 0.60 | *1.86* |
| 4A | Extracellular Glutamate HC | 3w | 0.18 | / |
| | | 6w | 0.18 | / |
| | | 12w | 0.26 | / |
| 4B | Extracellular Glutamate ADHD | 3w | 0.47 | / |
| | | 6w | 0.24 | / |
| | | 12w | 0.08 | / |
| 4C | Relative PSD95 Transcription | HC | 0.35 | / |
| | | ADHD | 0.29 | / |

| | | | | |
|---|---|---|---|---|
| | Relative vGLUT1 Transcription | HC | 0.55 | / |
| | | ADHD | 0.76 | / |
| | Relative vGLUT2 Transcription | HC | 0.43 | |
| | | ADHD | 0.58 | *1.96* |
| 4D | Relative EAAT1 Transcription | HC | 0.34 | / |
| | | ADHD | 0.42 | / |
| | Relative EAAT2 Transcription | HC | 0.32 | |
| | | ADHD | 0.59 | *1.56* |
| | Relative SNAT7 Transcription | HC | 0.21 | / |
| | | ADHD | 0.30 | / |
| 4E | PAX6 Relative Transcription | | | |
| | *NPCs* | HC | 0.07 | / |
| | | ADHD | 0.47 | / |
| | *2w CNs* | HC | 0.81 | / |
| | | ADHD | 0.37 | / |
| | *4w CNs* | HC | 0.72 | / |
| | | ADHD | 0.44 | / |
| 4F | vGLUT2 Relative Transcription | | | |
| | *2w CNs* | HC | 0.51 | / |
| | | ADHD | 0.26 | / |
| | *4w CNs* | HC | 0.23 | |
| | | ADHD | 0.53 | *2.92* |
| | *12w CNs* | HC | 0.40 | |
| | | ADHD | 0.48 | *2.26* |
| 5B | Mean Peak Amplitude | | | |
| | *8w CNs* | HC | 0.78 | / |
| | | ADHD | 0.22 | / |
| | *10w CNs* | HC | 0.38 | / |
| | | ADHD | 0.37 | / |
| | *12w CNs* | HC | 0.50 | / |
| | | ADHD | 0.22 | / |
| 5C | Max Peak Amplitude | | | |
| | *8w CNs* | HC | 0.35 | / |
| | | ADHD | 0.39 | / |
| | *10w CNs* | HC | 0.58 | / |
| | | ADHD | 0.53 | / |
| | *12w CNs* | HC | 0.51 | / |
| | | ADHD | 0.41 | / |
| 5E | Mean No. Peaks | | | |
| | *10w CNs* | HC | 0.24 | / |
| | | ADHD | 0.24 | / |
| | *12w CNs* | HC | 0.17 | |
| | | ADHD | 0.34 | *2.09* |
| 5E | Max No. Peaks | | | |
| | *10w CNs* | HC | 0.16 | |
| | | ADHD | 0.40 | *2.07* |

| | | | | |
|---|---|---|---|---|
| | *12w CNs* | HC | 0.19 | |
| | | ADHD | 0.13 | *1.78* |
